# Neuromyelitis optica spectrum disorder presenting as rapidly progressive dementia: A case report

**DOI:** 10.1097/MD.0000000000051464

**Published:** 2026-11-06

**Authors:** Daiping Hua, Shang Xiang, Lanting Sun, Liang Hong, Wenming Yang, Han Wang

**Affiliations:** a Department of Neurology, The First Affiliated Hospital of Anhui University of Chinese Medicine, Hefei, China; b Department of Neurology, The Fifth Affiliated Hospital of Anhui University of Chinese Medicine, Lu’an, China; c Key Laboratory of Xin’an Medicine, Ministry of Education, Hefei, China.

**Keywords:** aquaporin-4, case report, hyponatremia, neuromyelitis optica spectrum disorder, rapidly progressive dementia

## Abstract

**Rationale:**

Neuromyelitis optica spectrum disorder (NMOSD) is a rare autoimmune astrocytopathy primarily affecting the optic nerves and spinal cord. Diencephalic involvement is commonly detected on neuroimaging but often remains clinically silent. Rapidly progressive dementia (RPD) as the initial and primary manifestation of NMOSD is exceedingly rare.

**Patient concerns:**

A 64-year-old woman presented with a 10-day history of progressive cognitive slowing and memory decline. On admission, she was disoriented, apathetic, and unable to recognize her family members, with a Mini-Mental State Examination (MMSE) score of 3/30. Laboratory investigations revealed severe hyponatremia (serum sodium 112.60 mmol/L). Brain magnetic resonance imaging showed abnormal signal intensities involving the bilateral basal ganglia, hypothalamus, and mammillary bodies.

**Diagnoses:**

Autoimmune encephalitis, infectious encephalitis, Wernicke encephalopathy, and prion disease were initially considered and systematically excluded. Serum and cerebrospinal fluid testing revealed positive aquaporin-4 (AQP-4) antibodies with titers of 1:32 and 1:16, respectively. Based on the 2015 International Panel for NMO Diagnosis criteria, a final diagnosis of AQP-4 antibody-positive NMOSD was established.

**Interventions:**

The patient received intravenous methylprednisolone pulse therapy (1000 mg/d for 3 days, followed by a stepwise tapering regimen) and gradual correction of electrolyte disturbances.

**Outcomes:**

After 4 weeks of treatment, the patient showed marked cognitive improvement, with the MMSE score increasing from 3 to 18. At the 6-month follow-up, her clinical symptoms had almost completely resolved, the MMSE score had improved to 27/30, electrolyte levels had normalized, and follow-up magnetic resonance imaging demonstrated a significant reduction in lesion size with mild enlargement of the third ventricle.

**Lessons:**

This case demonstrates that AQP-4 antibody-positive NMOSD can present as a reversible RPD, potentially mediated by AQP-4 antibody-associated inflammatory injury of the diencephalon. It underscores that in patients with unexplained RPD – particularly when neuroimaging reveals lesions in AQP-4-enriched regions such as the hypothalamus, mammillary bodies, or periventricular areas surrounding the third ventricle, or when otherwise unexplained hyponatremia is present – NMOSD should be included in the differential diagnosis. Timely AQP-4 antibody testing should be pursued to facilitate early immunotherapy and optimize clinical outcomes.

## 1. Introduction

Neuromyelitis optica spectrum disorder (NMOSD) is a rare, humoral immune-mediated inflammatory demyelinating disease of the central nervous system. It predominantly manifests as optic neuritis and longitudinally extensive transverse myelitis, with a pathogenesis fundamentally linked to aquaporin-4 (AQP-4) antibody.^[1]^ Although diencephalic involvement is frequently detected on neuroimaging, such lesions may remain clinically asymptomatic.^[2]^ Cognitive deficits in NMOSD are increasingly recognized and encompass a broad spectrum, including impairments in attention, processing speed, and episodic memory, which are typically subacute to chronic in onset. Consequently, NMOSD presenting with an isolated diencephalic syndrome is rarely reported in the literature, and cases dominated by rapidly progressive dementia (RPD) as the primary manifestation are exceedingly uncommon. Herein, we describe a unique case of NMOSD presenting as RPD. This report aims to broaden the recognized clinical spectrum of the disease and heighten clinicians’ awareness of its atypical manifestations.

## 2. Case presentation

A 64-year-old female farmer presented to our neurology department on June 22, 2025, with a 10-day history of progressive cognitive slowing and memory decline. Thirteen days before the current admission, she had been hospitalized at a local hospital for slurred speech. Acute cerebral infarction was suspected, and antithrombotic and lipid-lowering therapies were initiated. However, over the following several days, her son observed a rapid deterioration in her cognitive function to the extent that she was no longer able to recognize her husband or son. She was therefore transferred to our department for further diagnostic evaluation of cognitive impairment. Throughout the disease course, the patient experienced no loss of consciousness, seizures, headaches, nausea, or vomiting. She also denied any history of fever, diarrhea, or vaccinations in the month preceding symptom onset. Upon admission, she exhibited prominent psychomotor retardation, hypophonia, and severe memory deficits. Her appetite was mildly decreased, but bowel and bladder functions remained intact. Her past medical history was notable for a 5-year history of hypertension (with a maximum recorded blood pressure of 160/80 mm Hg), for which she took amlodipine besylate 5 mg daily without regular blood pressure monitoring. She denied any history of occupational toxic exposures, infectious diseases (such as hepatitis or tuberculosis), major trauma, blood transfusions, or drug allergies.

On admission, the patient’s vital signs were stable. She was alert but apathetic, with psychomotor slowing and limited cooperation with motor commands. Cognitive assessment revealed global cognitive impairment involving orientation and both recent and remote memory. Her calculation ability was severely impaired, and she was unable to perform simple subtraction tasks. The pupils were equal, round, and reactive to light. Extraocular movements were full in all directions, with no nystagmus. The bilateral nasolabial folds were symmetric, the tongue protruded in the midline, and the gag reflex was preserved. Motor examination showed normal muscle tone in all 4 limbs, Medical Research Council grade 5/5 strength throughout. Gross examination of superficial and deep sensation was intact. Bilateral Babinski signs were observed. Finger-to-nose and heel-to-shin testing could not be performed because of poor cooperation. There were no signs of meningeal irritation. The Mini-Mental State Examination (MMSE) score was 3/30.

Laboratory investigations, including a complete blood count, urinalysis, stool analysis, liver and renal function tests, cardiac enzyme panel, and coagulation profile, were all within normal limits. The serum sodium level was 112.60 mmol/L. The lipid profile and C-reactive protein level were unremarkable. Given that severe hyponatremia may contribute to cognitive impairment, gradual sodium correction was initiated to minimize the risk of osmotic demyelination syndrome, with close monitoring of serum electrolyte levels. Further investigations were undertaken to identify the cause of the hyponatremia. Repeat electrolyte testing showed that the serum sodium level had increased to 135.16 mmol/L, while the chloride level was 87.52 mmol/L. However, the patient’s cognitive impairment persisted despite correction of the hyponatremia, with an MMSE of 4. Adrenocorticotropic hormone, thyroid function, and thyroid-related antibody levels were within normal limits. Tumor markers, an 8-item immune panel, antinuclear antibody profile, and vasculitis antibody panel were all negative. Ultrasonography of the kidneys and adrenal glands revealed no abnormalities.

Brain magnetic resonance imaging (MRI) revealed abnormal signal intensities involving the bilateral basal regions, hypothalamus, and mammillary bodies (Fig. 1). Although these findings were suggestive of Wernicke encephalopathy, the serum vitamin B1 level was normal. Further history-taking confirmed that the patient had previously been in good health, maintained a regular diet, and had no history of malnutrition or alcohol consumption. Wernicke encephalopathy was therefore considered unlikely.

**Figure 1.**
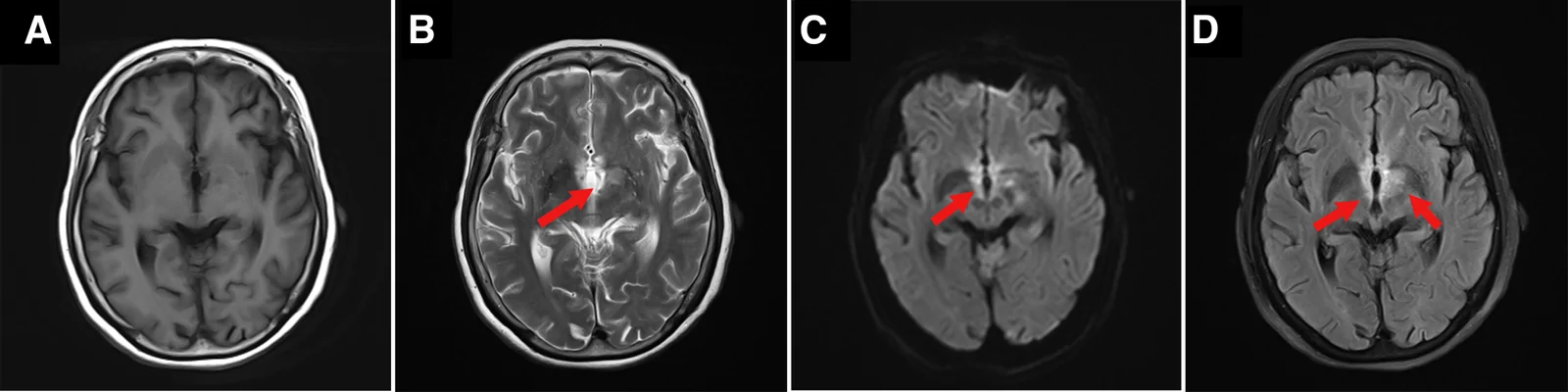
Initial cranial MRI scan. Red arrows indicate abnormal signals in the bilateral basal ganglia and hypothalamus. MRI = magnetic resonance imaging.

A lumbar puncture was subsequently performed to further clarify the etiology. The cerebrospinal fluid (CSF) opening pressure was 120 mmH_2_O. CSF analysis showed a white blood cell count of 29 × 10^6^/L, comprising 9% polymorphonuclear cells and 91% mononuclear cells. The red blood cell count was 0, and the Pandy test was negative. CSF biochemical parameters were within normal limits, and testing for autoimmune encephalitis antibodies was negative. Serum and CSF samples were then tested for demyelinating disease-associated antibodies using a live cell-based assay. The AQP-4 antibody titer was 1:32 in serum and 1:16 in CSF. Visual evoked potentials, electroencephalography, and MRI of the cervical, thoracic, lumbar, and sacral spinal cord showed no abnormalities. Based on the diagnostic criteria established by the International Panel for NMOSD,^[3]^ a final diagnosis of NMOSD was made.

The patient received intravenous methylprednisolone pulse therapy according to the following regimen: 1000 mg once daily for 3 days, followed by 500 mg once daily for 3 days, 240 mg once daily for 3 days, and then 120 mg once daily for 3 days. Treatment was subsequently switched to oral prednisone at 60 mg once daily for 7 days, followed by 50 mg once daily for 7 days. The dose was then gradually tapered to a moderate dose of 40 mg/d and further reduced by 5 mg every 2 weeks until a maintenance dose of 10 to 15 mg once daily was reached. Supportive treatment included maintenance of fluid and electrolyte balance, stress ulcer prophylaxis, and calcium supplementation. After 4 weeks of treatment, at the time of discharge, the patient showed marked improvement in bradyphrenia and memory impairment compared with admission. The MMSE score had increased to 18, and she was able to recognize most of her relatives and friends. At the 6-month outpatient follow-up, her clinical symptoms had almost completely resolved (Fig. 2). Electrolyte levels had returned to normal, and the MMSE score had improved to 27. Follow-up brain MRI showed mild enlargement of the third ventricle and a marked reduction in the abnormal T2/FLAIR hyperintensities in the bilateral basal ganglia and hypothalamus compared with baseline, although some residual signal abnormality persisted (Fig. 3). At the 6-month follow-up, the patient was maintained on oral prednisone at a tapering dose of 10 to 15 mg/d; no additional steroid-sparing immunosuppressants had been initiated due to the sustained clinical improvement.

**Figure 2.**
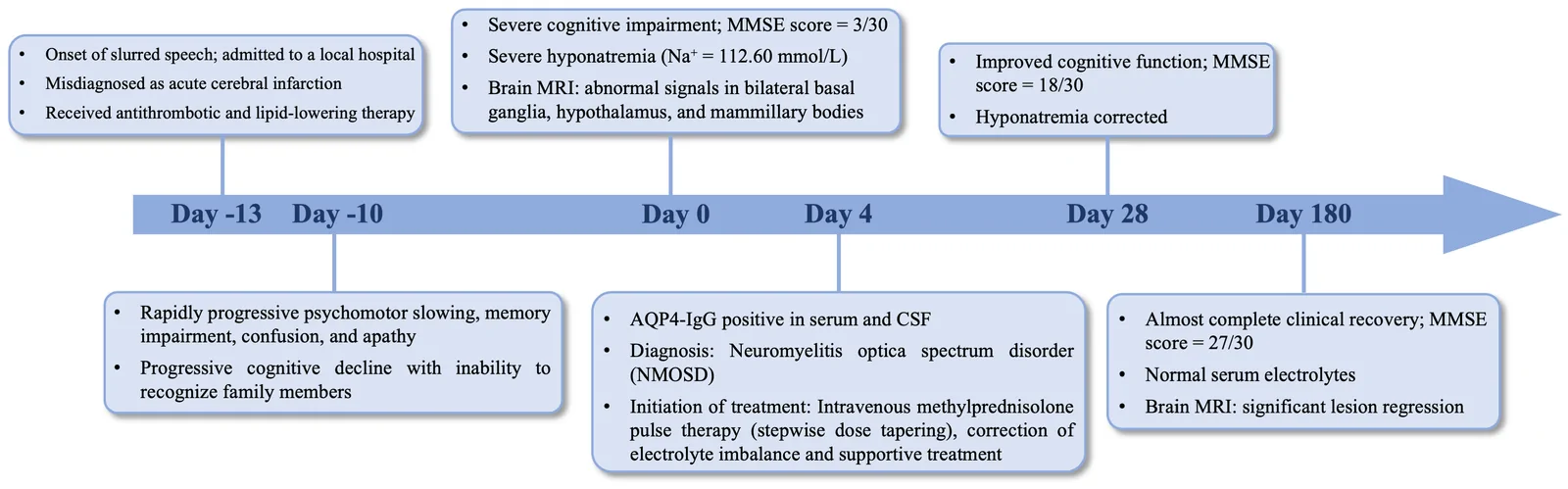
Patient clinical course timeline. AQP4-IgG = aquaporin-4 immunoglobulin G, CSF = cerebrospinal fluid, MMSE = Mini-Mental State Examination, MRI = magnetic resonance imaging, NMOSD = neuromyelitis optica spectrum disorder.

**Figure 3.**
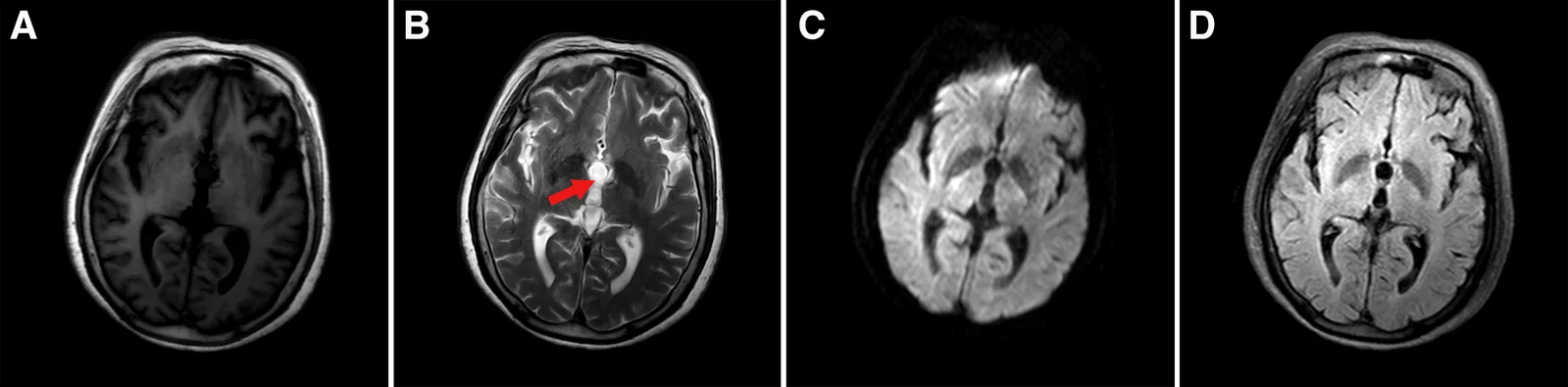
Follow-up brain MRI scan. (A) T1-weighted image; (B) T2-weighted image; (C) FLAIR image. The red arrow points to the mild enlargement of the third ventricle. FLAIR = fluid-attenuated inversion recovery, MRI = magnetic resonance imaging.

## 3. Discussion

NMOSD is an autoimmune disease of the central nervous system characterized by AQP-4 antibody-mediated astrocytic injury. Its typical clinical phenotypes include optic neuritis, acute myelitis, area postrema syndrome, brainstem syndrome, diencephalic syndrome, and cerebral syndrome.^[4]^ RPD is a rare and atypical presentation of NMOSD.^[5]^ In the absence of typical optic neuritis or myelitis, such cases may be misdiagnosed as cerebrovascular disease, infectious encephalitis, autoimmune encephalitis, metabolic encephalopathy, or prion disease.^[6,7]^ In this case, the patient developed rapidly progressive cognitive decline, psychomotor slowing, apathy, and disorientation. Brain MRI showed lesions mainly involving the bilateral basal ganglia, hypothalamus, and mammillary bodies, and AQP-4 antibodies were detected in both serum and CSF. The marked clinical and radiological improvement after corticosteroid pulse therapy supported a diagnosis of NMOSD-related diencephalic involvement presenting as rapidly progressive cognitive impairment. This case highlights NMOSD as a potentially treatable cause of RPD.^[8]^

From a pathophysiological perspective, AQP-4 is widely expressed on astrocytic endfeet, with particularly high density in periventricular regions, the hypothalamus, area postrema, periaqueductal gray matter, and the corpus callosum. This distribution pattern underlies the anatomical selectivity of brain lesions in NMOSD.^[9]^ The mechanism of cognitive impairment in NMOSD is primarily attributed to limbic system involvement. The precise pathophysiological mechanisms by which diencephalic lesions lead to cognitive impairment in NMOSD remain incompletely understood, and functional neuroimaging or pathological data would have been valuable in elucidating these mechanisms; however, such data were not available for this patient. The hypothalamus, mammillary bodies, and fornix constitute critical components of the Papez circuit. In the present case, MRI-demonstrated lesions in the hypothalamus and mammillary bodies directly disrupted signal transmission within this memory circuit, resulting in acute or subacute cognitive decline resembling Alzheimer’s disease or autoimmune encephalitis.^[10,11]^ The diencephalon, particularly the hypothalamus and periventricular structures surrounding the third ventricle, is rich in AQP-4 expression. Consequently, involvement of these regions can manifest as somnolence, endocrine dysfunction, thermoregulatory abnormalities, and fluid-electrolyte disturbances.^[12]^

RPD is a syndrome characterized by progressive cognitive decline over days to months, with a broad etiologic spectrum that includes prion disease, autoimmune encephalitis, toxic-metabolic encephalopathy, infectious encephalitis, vascular disorders, neoplasms, and demyelinating diseases.^[13]^ In clinical practice, diagnosis may be delayed when cognitive decline, apathy, and memory impairment predominate in the absence of typical NMOSD features, such as visual loss, limb weakness, or intractable hiccups and vomiting. The present patient was initially diagnosed with cerebral infarction; however, the subsequent clinical course was inconsistent with cerebrovascular disease. Her symptoms continued to worsen despite treatment for cerebral infarction, and MRI lesions involving the bilateral basal ganglia, hypothalamus, and mammillary bodies did not conform to a typical vascular territory. CSF analysis showed mild inflammatory changes. In addition, MRI abnormalities in the hypothalamus and mammillary bodies, along with marked hyponatremia, suggested hypothalamic–pituitary axis involvement, potentially leading to dysregulated antidiuretic hormone secretion or impaired osmoregulation. The patient met the diagnostic criteria for the Syndrome of Inappropriate Antidiuretic Hormone Secretion^[14]^: serum sodium was 112.60 mmol/L (<135 mmol/L), serum osmolality was 245 mOsm/kg (<275 mOsm/kg), urine osmolality was 486 mOsm/kg (>100 mOsm/kg), and urine sodium was 68 mmol/L (>40 mmol/L), with no evidence of hypovolemia, adrenal insufficiency, or diuretic use. Cerebral salt wasting was considered but deemed unlikely due to the absence of volume depletion and the presence of concentrated urine.^[15]^ Hyponatremia itself may also aggravate consciousness disturbance and cognitive impairment.^[16]^ To avoid central pontine myelinolysis, serum sodium was corrected gradually; however, cognitive impairment persisted after correction of the electrolyte disturbance. Moreover, hyponatremia alone could not explain the abnormal signals in the basal ganglia and mammillary bodies. Wernicke encephalopathy was also considered and ruled out based on normal serum vitamin B1 levels and dietary history.^[17]^ The diagnosis of NMOSD was ultimately confirmed by demyelinating antibody testing. Therefore, in patients with unexplained hyponatremia and lesions involving the basal ganglia, hypothalamus, mammillary bodies, or other periependymal regions – particularly when accompanied by somnolence or neuropsychiatric abnormalities – NMOSD should be included in the differential diagnosis, and AQP-4 antibody testing should be actively pursued.

Regarding differential diagnosis, autoimmune encephalitis was the primary consideration in this case. Autoimmune encephalitis typically presents with recent memory impairment, neuropsychiatric abnormalities, and seizures. MRI commonly involves the medial temporal lobe, hippocampus, and amygdala. In contrast, the lesions in our patient were predominantly located in the hypothalamus and mammillary bodies, without seizures or typical imaging features of limbic system involvement, which does not fully support classic autoimmune encephalitis.^[18,19]^ Infectious encephalitis was another differential consideration. The patient lacked fever, headache, seizures, or obvious meningeal irritation signs. CSF analysis showed only mild leukocytosis with normal protein levels, and there were no diffuse or temporal lobe-predominant involvement patterns commonly observed in infectious encephalitis.^[20]^ Creutzfeldt–Jakob disease was also considered in the differential diagnosis. Although Creutzfeldt–Jakob disease can present with RPD, it is typically accompanied by myoclonus, extrapyramidal symptoms, characteristic electroencephalography changes, and cortical ribboning or basal ganglia hyperintensity on diffusion-weighted imaging. The rapid improvement following corticosteroid therapy in this case is clearly inconsistent with the natural history of prion disease.^[13]^

Regarding treatment and prognosis, high-dose corticosteroid pulse therapy during the acute phase, with plasma exchange when necessary, represents the first-line approach for controlling acute NMOSD attacks.^[21,22]^ Following confirmation of the diagnosis, this patient promptly received stepwise corticosteroid pulse therapy followed by sequential oral tapering for maintenance. With aggressive immunosuppressive treatment and correction of fluid-electrolyte disturbances, the patient’s cognitive function showed marked improvement. Follow-up MRI at 6 months demonstrated significant absorption and reduction of lesions, suggesting that RPD secondary to diencephalic involvement may have a favorable prognosis when early, accurate diagnosis and intervention are achieved. Although the patient showed sustained remission on tapering prednisone at 6 months, long-term maintenance therapy with steroid-sparing immunosuppressive agents such as rituximab, mycophenolate mofetil, or azathioprine may still be necessary to prevent future relapses. The decision to initiate such agents should be individualized, and close monitoring during extended follow-up is essential.

## 4. Conclusion

This report describes a case of AQP-4 antibody-positive NMOSD presenting predominantly as RPD. The patient developed progressive cognitive decline accompanied by severe hyponatremia. Brain MRI revealed lesions involving the bilateral basal ganglia, hypothalamus, and mammillary bodies. After corticosteroid pulse therapy, both cognitive impairment and radiological abnormalities improved markedly, supporting NMOSD as a treatable cause of RPD. Several limitations should be acknowledged. This is a single-case report. Functional neuroimaging and pathological data were not available, limiting further exploration of the mechanisms underlying neural injury in NMOSD-related cognitive impairment. Future multicenter studies with larger cohorts are warranted to further define the clinical spectrum and optimal management strategies for such atypical NMOSD presentations. In addition, the follow-up period was relatively short (6 months), limiting conclusions regarding long-term cognitive outcomes, relapse risk, and the optimal maintenance treatment strategy; extended follow-up will be necessary to better define these parameters. In conclusion, clinicians should maintain a high index of suspicion for NMOSD in patients presenting with unexplained RPD, diencephalic lesions, and electrolyte disturbances. Further studies are warranted to better define the clinical spectrum, pathophysiological mechanisms, and standardized diagnostic and therapeutic strategies for such atypical NMOSD presentations.

## Acknowledgments

The authors gratefully acknowledge the technical support and collaboration provided by the colleagues in the Department of Neurology, the First/Fifth Affiliated Hospital of Anhui University of Chinese Medicine.

## Author contributions

**Conceptualization:** Daiping Hua, Shang Xiang.

**Data curation:** Lanting Sun, Liang Hong.

**Writing – original draft:** Daiping Hua, Shang Xiang.

**Writing – review & editing:** Han Wang, Wenming Yang.
